# Rx and its downstream factor, Musashi1, is required for establishment of the apical organ in sea urchin larvae

**DOI:** 10.3389/fcell.2023.1240767

**Published:** 2023-08-15

**Authors:** Junko Yaguchi, Shunsuke Yaguchi

**Affiliations:** ^1^ Shimoda Marine Research Center, University of Tsukuba, Shimoda, Japan; ^2^ PRESTO, Japan Science and Technology Agency, Kawaguchi, Japan

**Keywords:** sea uchin, neurogenesis, serotonin, acetylcholine, neuroectoderm

## Abstract

Acetylcholine, a vital neurotransmitter, plays a multifarious role in the brain and peripheral nervous system of various organisms. Previous research has demonstrated the proximity of cholinergic neurons to serotonergic neurons in the apical organ of sea urchin embryos. While several transcription factors have been identified as playing a role in the development of serotonergic neurons in this region of a sea urchin, *Hemicentrotus pulcherrimus*, comparatively little is known about the specific transcription factors and their spatiotemporal expression patterns that regulate the development of cholinergic neurons. In this study, we establish the requirement of the transcription factor Rx for the development of cholinergic neurons in the apical organ of the species. Furthermore, we investigate the role of the RNA-binding protein Musashi1, known to be involved in neurogenesis, including cholinergic neurons in other organisms, and demonstrate that it is a downstream factor of Rx, and that choline acetyltransferase expression is suppressed in Musashi1 downregulated embryos. Our research also highlights the intricate network formed by neurons and other cells in and around the apical organ of sea urchin larvae through axons and dendrites, providing possibility for a systematic and complexed neural pattern like those of the brain in other organisms.

## Introduction

To gain insights into the nature of organisms, it is crucial to elucidate the organization and functioning of nervous systems, as they play a fundamental role in shaping behaviors. The development of sophisticated nervous systems throughout evolution has undoubtedly contributed to the efficiency and survival capabilities of animals. Notably, human beings possess one of the largest and most complex brains among all animals (Heldstab et al., 2022), leading to the development of an incredible array of communication tools such as conversation and the internet. Insects, too, exhibit intricate brains and display astonishing responses to environmental changes. Consequently, extensive research has been conducted, primarily focusing on mammals and insects, to unravel the mysteries surrounding brains and nervous systems.

However, the current body of knowledge regarding the characteristics of nervous systems in non-model organisms remains inadequate for meaningful comparisons with those of model organisms. This limitation hampers our ability to assess the extent of diversification in nervous systems during evolution.

Specifically, detailed studies on the nervous system organization in Ambulacraria, including echinoderms and hemichordates, have not been fully reported, similar to other non-model organisms, despite their significant position in the evolutionary tree (Burke et al., 2006; Angerer et al., 2011; Holland, 2015). While numerous studies have explored gene expression patterns during the embryonic and early developmental stages of these species, investigations into individual neuron patterns have been limited thus far (Buznikov et al., 2001; Nakajima et al., 2004; Arshinoff et al., 2022). For example, the development of serotonergic neurons in the anterior neuroectoderm of sea urchin embryos and larvae was initially described in the 1980s through immunochemical technologies (Bisgrove and Burke, 1986). Subsequently, other types of neurons have been identified using immunohistochemistry (Nakajima et al., 2004; Yaguchi et al., 2022). However, these studies have primarily focused on the main positions of neural cell bodies and provided only limited descriptions of individual neural fiber in embryos and larval stages. Consequently, detailed anatomical characteristics of the nervous systems in echinoderm larvae, particularly in the anterior neuroectodermal region, remain insufficiently explored. Given strong evidence suggesting that the serotonergic nervous system in the anterior neuroectoderm of sea urchin larvae integrates environmental signals into larval behaviors (Yaguchi and Yaguchi, 2021) and exhibits gene expression profiles similar to those of the forebrain in vertebrates (Wei et al., 2009; Angerer et al., 2011), the anterior neuroectoderm may be considered the brain in sea urchin larvae. Hence, in this paper, we refer to it as the “brain”.

To date, it has been reported that two main types of neurons, serotonergic and non-serotonergic, are present in the brain region of sea urchin larvae (Nakajima et al., 2004). Recent findings have identified the majority of non-serotonergic neurons as cholinergic in *Lytechinus variegatus* (Slota and McClay, 2018). Additionally, the presence of various peptidergic neurons in and around the brain has been reported using *in situ* hybridization and immunohistochemical techniques (Beer et al., 2001; Wood et al., 2018). While information regarding the developmental mechanisms of these nervous systems in the brain, involving transcription factors and signaling molecules, continues to accumulate, a more detailed analysis is necessary to understand the complete process of nervous system development, including specification, differentiation, and network formation. Therefore, our focus is on describing the process of neural construction in the larval brain of sea urchins and reporting a portion of the molecular mechanisms that regulate its formation, using the Western Pacific model sea urchin, *Hemicentrotus pulcherrimus*.

## Materials and methods

### Animal collection and embryonic/larval culture

Adult *Hemicentrotus pulcherrimus* were collected around Shimoda Marine Research Center, University of Tsukuba, and around the Marine and Coastal Research Center, Ochanomizu University. This species was collected under the special harvest permission of prefectures and Japan Fishery cooperatives. Gametes were collected by the intrablastocoelic injection of 0.5 M KCl, and the embryos/larvae of H. pulcherrimus was cultured at 15°C, in glass beakers or plastic dishes that contained filtered natural seawater (FSW) with 50 μg/mL kanamycin.

### Whole-mount *in situ* hybridization and immunohistochemistry

Whole-mount *in situ* hybridization was performed as described previously (Yaguchi and Katow, 2003) with some modifications. cDNA mix from several embryonic stages was used to make RNA probes based on the *H. pulcherrimus* genome and transcriptome (Kinjo et al., 2018). The samples were incubated with RNA probes for *foxQ2* (HPU_15608), *rx* (HPU_04689), *hbn* (HPU_04688), *lefty* (HPU_15030), *neurogenin* (HPU_07864), *choline acetyltransferase* (HPU_01496), *msi1* (HPU_03784) and *tryptophan 5-hydroxylase* (*tph*; HPU_21307) (Ayala et al., 2007) at a final concentration of 0.4–1.2 ng/μL at 50°C for 5 days. The probes were detected with the Tyramide Signal Amplification Plus System (TSA; Akoya Biosciences, Marlborough, MA, United States) or Alkaline phosphatase-based chromogenic system described previously (Erkenbrack et al., 2019).

Whole-mount immunohistochemistry was also performed as described previously. The samples were blocked with 1% skim milk in PBST for 1 h at RT and incubated with primary antibodies (dilutions: mouse anti-Synaptotagmin B (SynB) (Nakajima et al., 2004), 1:100; mouse anti-ChAT (9), 1:100; rabbit anti-serotonin (#S5545; Sigma-Aldrich), 1:1000) overnight at 4°C.

### Microinjection of morpholino anti-sense oligonucleotides (MO), mRNAs, and DNA

Microinjection was performed according to a previously described method (Yaguchi, 2019) with injection buffer (24% glycerol, 20 mM HEPES pH 8.0 and 120 mM KCl). The morpholino (Gene Tools, Philomath, OR, United States) sequences and the in-needle concentrations in injection buffer were as follows:

Rx-MO1 (1.9–3.8 mM): 5′- GGG​TGA​TGC​GCT​CCA​TCC​ATT​GTT​A -3′,

Rx-MO2 (1.0–1.9 mM): 5′- TTT​GTG​ACT​GAT​CGT​CTT​TCC​AAA​C -3′,

Msi1-MO1 (0.5–1.0 mM): 5′- AAC​CCT​CAA​CTA​AAA​AGG​CCC​AAT​A-3′,

Msi1-MO2 (1.9 mM): 5′- GAA​TTG​GCA​AAC​GGT​CCT​TCT​TAA​C-3′,

and Hbn-MO1 (0.7 mM): 5′- AAA​ATG​AAC​GGA​ACA​AGT​CCA​GTG​T -3’.

(previously characterized) (Yaguchi and Yaguchi, 2019).

Two non-overlapping translation-blocking morpholinos for Rx and Musashi were used to confirm the specificity of their function (Supplementary Figures S1, S2). For negative control experiments, we injected random MO (2.0 mM: Gene Tools, Supplementary Figure S1) or injection buffer only.

### Microscopy and image analysis

Live samples were observed under a light/fluorescence microscope (IX70, Olympus, Tokyo, Japan). The fixed and stained specimens were observed using a light/fluorescence microscope (IX70, Olympus) and a confocal laser scanning microscope (FV10i, Olympus). All transmission images were taken with an IX70 microscope. The Figure panels and drawings for the Figures were made using Adobe Photoshop and Microsoft PowerPoint.

## Results

### Developmental anatomy of brain neurons in sea urchin larvae

**FIGURE 1:**
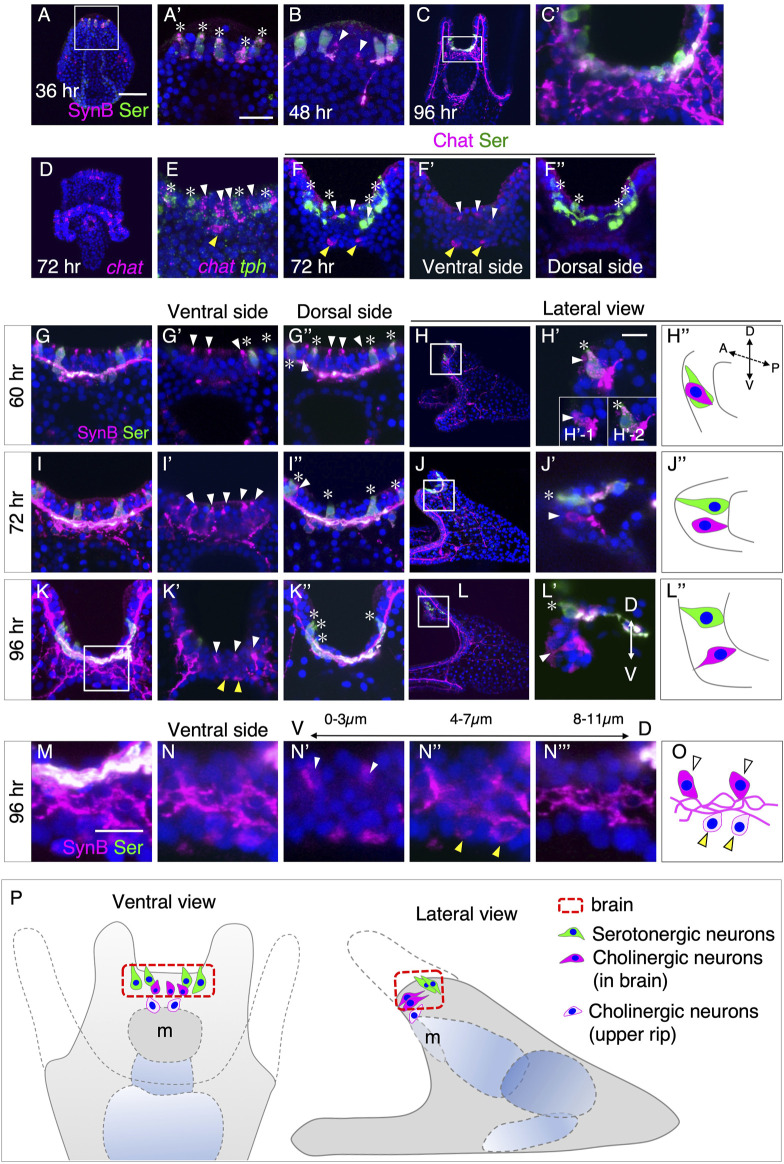
Spatiotemporal pattern of serotonergic and cholinergic neuron in the brain. **(A,A′)** Expression of serotonin (asterisks) begins around at 36 h prism larva in the brain. **(A′)** Magnified image of the square in **(A)**. **(B)** Expression of non-serotonergic neurons (Serotonin -/SynB +, arrowheads) begins at 48 h pluteus larva in the brain. **(C,C′)** Expression pattern of Serotonin and SynB at 96 h **(C′)** A magnified image of the rectangle in **(C)**. **(D)** *chat* mRNA expression pattern at 72 h pluteus larva. **(E)** *chat* (white arrowheads) and *tph* (asterisks) expression patterns at 72 h pluteus larva in the brain. **(F–F′′)** Chat (white arrowheads) and Serotonin (asterisks) expression pattern in brain. **(F′,F′′)** are ventral and dorsal side of **(F)**, respectively. Yellow arrowheads in **(E,F,F′)** show cholinergic neurons in the edge of the upper lip. There are only Chat positive cells at ventral side and there are only serotonergic neurons at dorsal side. **(G–L)** Serotonin (asterisks) and non-serotonergic neurons (white arrowheads) expression pattern at 60 h, 72 h and 96 h **(G′)**, **(I′)** and **(K′)** are ventral side images of **(G)**, **(I)** and **(K)**, respectively, and **(G′′)**, **(I′′)** and **(K′′)** are dorsal side images of **(G)**, **(I)** and **(K)**, respectively. **(H′)**, **(J′)** and **(L′)** are magnified images of squares in **(H)**, **(J)**, and **(L)**, respectively. **(H′′)**, **(J′′)** and **(L′′)** are schematic images of **(H′)**, **(J′)** and **(L′)**, respectively. **(H′)** is a stacked image of 8 sections (1 µm interval) and the insertion diagrams of **(H′)**, **(H′-1)** and **(H′-2)** show stacked images of the front and back 4 sections, respectively. These images show that there is a non-serotonergic neuron in **(H′-1)**, and, on the other hand, there is a serotonergic neuron in **(H′-2)**. A, anterior. P, posterior. D, dorsal. V, ventral. **(M–O)** The detailed expression pattern of non-serotonergic neurons in brain (white arrowheads) and in the edge of the upper lip (yellow arrowheads). **(M)** A magnified image of the square in **(K)**. **(N)** A stacked image of ventral side of **(M)** (12 sections). A stacked image of the most ventral 4 sections **(N′)**, the middle 4 sections **(N′′)** and the most dorsal 4 sections **(N‴)**. **(O)** A schematic image of **(N)**. **(P)** Schematic image of neurons in brain and the edge of the upper lip. Bars in **(A)** and **(A′)** are 50 µm and 20 μm, respectively. Bars in **(H)** and **(M)** are 10 µm.

Previous research has demonstrated the presence of non-serotonergic neurons in the brains of sea urchin larvae (Nakajima et al., 2004). In the species *L. variegatus*, these neurons were found to be cholinergic (Slota and McClay, 2018). In *H. pulcherrimus*, one of the commonly studied sea urchins in the Western Pacific, serotonergic neurons (serotonin+/synaptotagminB [SynB]+) appear in the brains of prism larvae at 36 h (hr) (Figures 1A, A′). Non-serotonergic neurons (serotonin-/SynB+) begin to emerge in the early pluteus larvae (Figure 1B). The number of these non-serotonergic neurons increases as development progresses (Figures 1C, C').

To verify whether these non-serotonergic neurons in *H. pulcherrimus* are also cholinergic, similar to *L. variegatus*, we detected the messenger RNA (mRNA) of choline acetyltransferase (ChAT) and tryptophan 5-hydroxylase (TPH), a rate-limiting enzyme of serotonin synthesis, in the larvae and examined the details using a confocal microscope. Besides the previously reported expression of *chat* in ciliary band neurons in *H. pulcherrimus* larvae (Yaguchi et al., 2022), *chat* signals were also detected in the brain region at 72 h (Figures 1D, E), as observed in *L. variegatus* (Slota and McClay, 2018). The presence of ChAT protein in these neurons was confirmed using specific antibodies (Figures 1F–F″).

Additionally, we observed the presence of one to three cholinergic neurons at the edge of the upper lip of the larval mouth, distinct from the brain neurons (yellow arrowheads in Figure 1F, F′). However, due to the complexity of axons and/or dendrites in the brain and mouth regions, tracing the precise spatial patterns of cholinergic neurons and their connections with other neurons in and around the brain region was challenging (Figures 1C, C'). Therefore, we conducted a more precise investigation of the spatiotemporal patterns of cholinergic neurons by observing neurogenic markers during the early pluteus stages. Since Synaptotagmin B (SynB) represents all neurons and their axons/dendrites, and most SynB-neurons at this developmental stage are cholinergic, except for serotonergic neurons in the brain (Figures 1D, E), we initially used Serotonin and SynB antibodies to elucidate the neural patterns at 60 h, 72 h, and 96 h larvae, with a particular focus on the brain and upper lip region.

In 60-h early pluteus larvae, cholinergic neurons were found to be located near serotonergic neurons (Figures 1G–G″). A lateral view, providing a longitudinal optical section, revealed that cholinergic and serotonergic neurons aligned in a single row along the left-right body axis in the brain region (Figures 1H–H″). By 72 h pluteus larvae, the majority of cholinergic neurons appeared to have shifted more towards the ventral side rather than the dorsal side (Figures 1I–I''), which was supported by the lateral view (Figures 1J–J″). This ventral shift of cholinergic neurons became even more pronounced by 96 h pluteus larvae, resulting in the detection of cholinergic neurons only on the ventral side and serotonergic neurons exclusively on the dorsal side (Figures 1K–K″). Detailed observations of optical sections from ventral to dorsal side using a confocal microscope (Figures 1L–L″) revealed that the tips of cholinergic neurons leaned towards the ventral direction, with a complex of neural processes located at the basal side of the epithelial layer (Figures 1M–N‴).

In addition to the cholinergic neurons in the brain (Figure 1N', white arrowheads), cholinergic neurons in the upper lip (Figure 1N'', yellow arrowheads) extended their processes towards the basal complex (Figures 1N''', O). Figure 1P summarizes the patterning of brain serotonergic and cholinergic neurons, as well as the upper lip cholinergic neurons, in 4-day larvae. Based on our observations, two sets of neurons are arranged in the brain of sea urchin larvae: serotonergic neurons on the dorsal side and cholinergic neurons on the ventral side in *H. pulcherrimus*. Initially, both types of neurons are situated in a single row on the dorsal side of the larvae, and cholinergic neurons appear to shift towards the ventral side during development.

### Rx is required for development of cholinergic neurons in sea urchin brains

Retinal homeobox (Rx) is a specific transcription factor expressed in the prospective brain region during early development and in some neural progenitor cells in other sea urchin species (Burke et al., 2006; Yaguchi et al., 2012). However, its function remains unknown, although a previous study has suggested a role for Rx in the differentiation of serotonergic neurons based solely on its expression pattern (Wei et al., 2009). Given the importance of Rx in brain formation in other organisms, we hypothesize that it plays a crucial role in sea urchin brain specification. Therefore, we investigated the expression and function of Rx in *H. pulcherrimus*.

**FIGURE 2:**
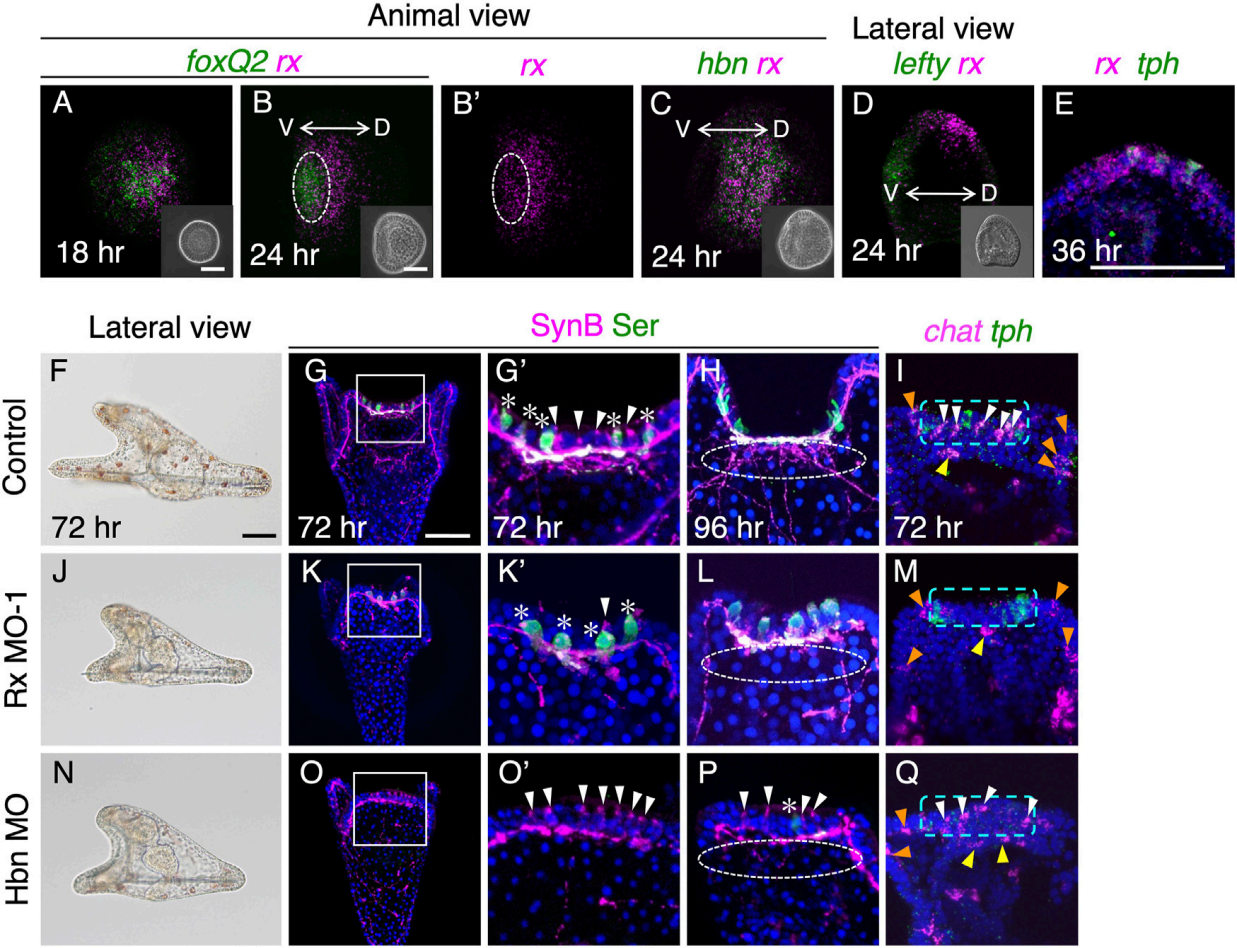
Rx is required for development of cholinergic neurons. **(A,B)** *foxQ2* and *rx* expression patterns at 18 h and 24 h. White dotted circle in **(B,B′)** show *foxQ2* positive region. **(C)** *hbn* and *rx* expression patterns in 24 h **(D)** *lefty* and *rx* patterns in 24 h. The *rx* expressing region shift to dorsal side. The inserts in A-D are bright field images. V, ventral, D, dorsal. **(E)** *rx* and *tph* are co-expressed in brain region of 36 h larvae. **(F,J,N)** Bright field images viewed from lateral in control, Rx MO-1 and Hbn MO injected embryos. **(G,G′,K,K′,O,O′)** Almost all non-serotonergic neurons (cholinergic neurons; white arrowheads) are disappeared in the brain of Rx morphants, whereas in Hbn morphants, serotonergic neurons (asterisks) are mostly disappeared. **(G′,K′,O′)** Magnified images of the squares in **(G)**, **(K)** and **(O)**. **(H,L,P)** Neuro-plexus from cholinergic neurons are disappeared in both Rx and Hbn morphants (white dotted lined circles). **(I,M,Q)** There are few *chat* positive cells (white arrowheads) at brains in Rx morphant, but there are in Hbn morphants as same as controls. Green dotted rectangles show brain region. *Chat* cells in upper lips (yellow arrowheads) and ciliary bands (orange arrowheads) are invariant in control and these morphants. Bars in insets of **(A)** and **(B)**, and **(F)** and **(G)**, are 50 µm.

At 18 h in mesenchyme blastulae, mRNA of *rx* was found to be co-expressed with *foxQ2* (Figure 2A). As FoxQ2 is known to be a regional specifier for brain regions in sea urchin embryos/larvae, the co-expression of *foxQ2* and *rx* strongly suggests that FoxQ2 regulates the expression of *rx*. Indeed, when we attenuated FoxQ2 function using morpholino antisense oligonucleotide (MO), the expression of *rx* was completely abolished in the brain region (Supplementary Figures S3A, B). While the *foxQ2* expression region gradually becomes restricted to the anterior end of embryos as development progresses (Tu et al., 2006; Yaguchi et al., 2008), the *rx* region remains not only at the anterior end but also in the more dorsal region, similar to the expression pattern of *homeobrain* (*hbn*) at 24 h in early gastrula (Figures 2B–D) (Yaguchi and Katow, 2003). We detected *rx* signal in cells that also expressed *tryptophan 5-hydroxylase* (*tph*) at 36 h in larvae (Figure 2E), but the *rx* signal disappeared in *tph*-positive cells by 48 h (see Supplementary Figure S5I). Since 36 h is close to the onset of serotonin synthesis (Yaguchi et al., 2000; Ayala et al., 2007), it is plausible that Rx is involved in the differentiation of serotonergic neurons in *H. pulcherrimus*.

To investigate the precise functions of Rx in the development of serotonergic neurons, we conducted knockdown experiments by injecting Rx-MO. As a result, we did not observe any disturbances in the development of serotonergic neurons in Rx morphants (Figures 2K–M). On the other hand, the number of brain cholinergic neurons was significantly reduced in these morphants (Figures 2K'–M, white arrowhead) compared to controls (Figure 2G') (Supplementary Table S1). Larvae injected with a random morpholino as a control did not show any effects on the development of cholinergic neurons (Supplementary Figures S1C, D). Moreover, Rx-MO2 morphants exhibited identical phenotypes to Rx-MO1 morphants, providing further support for the requirement of Rx in the development of cholinergic neurons in the brain. Strikingly, this phenotype is completely opposite to that of Hbn morphants, in which serotonergic neurons are absent while cholinergic neurons remain (Figures 2N–Q) (Yaguchi and Katow, 2003), despite the almost identical expression patterns of both genes. Rx morphants displayed shorter arms than controls, resembling the phenotype of Hbn morphants (Figures 2F, J, N). Our observations confirmed that the remaining non-serotonergic neurons in the brain region of Hbn morphants were cholinergic (Figures 2O–Q). Notably, cholinergic neurons at the edge of the upper lip and in the non-brain ciliary band region were barely affected in both Rx and Hbn morphants (yellow and orange arrowheads in Figures 2I, M, Q).

Although *rx* and *hbn* are co-expressed during early stages (Figure 2C), it is likely that these transcription factors target different genes, at least in the development of sea urchin larval brains. In a previous study using *L. variegatus*, it was reported that the neurogenin ortholog (Lv-ngn) is expressed throughout the ciliary band, including the brain, and *Lv-ngn* is necessary for the specification of cholinergic neurons in the ciliary band (Slota and McClay, 2018). We also detected the expression of *Hp-ngn* throughout the ciliary band and brain from 48 h to 60 h in *H. pulcherrimus* (Supplementary Figures S4A–D). In early pluteus larvae of *H. pulcherrimus*, the *ngn*-positive cells in the brain express *chat* but not *tph*, similar to *L. variegatus* (Supplementary Figure S4E–E‴, F–F‴).

### Musashi1 is a downstream factor of Rx

**FIGURE 3:**
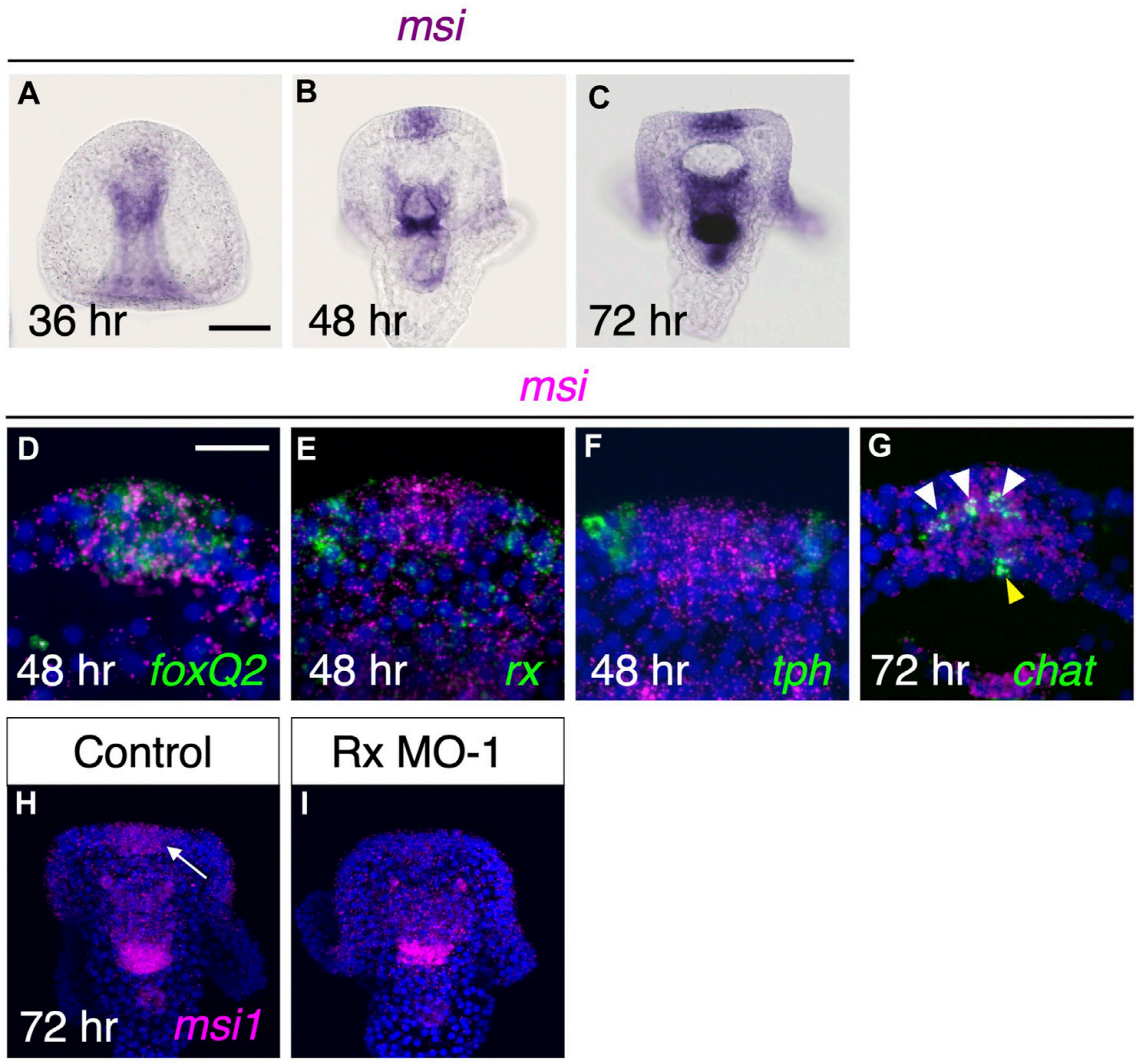
Msi is downstream factor of Rx during brain formation. **(A–C)** In brain region, *msi* begins to express by 48 h pluteus larvae. **(D,E,F)** *msi* is co-expressed with *foxQ2* but neither *rx* nor *tph* at 48 h **(G)** msi express at *chat* expressing region in the brain at 72 h. White arrowheads and yellow arrowhead show cholinergic neurons in brain and at upper lip region, respectively. **(H,I)** *msi* in brain (arrow) is missing in Rx morphants.

Musashi is an RNA binding protein that is expressed in neural progenitor cells and plays a role in the development of the brain and central nervous system in various organisms (e.g.,25,26). Previous studies have suggested that Musashi is involved in the differentiation of cholinergic neurons, as it is co-expressed with *chat* in these organisms (Sakakibara et al., 2001; Higuchi et al., 2008; Perry et al., 2012). Sea urchins also possess a Musashi1-like gene (referred to as Msi1) in their genomes (Sodergren et al., 2006; Kinjo et al., 2018; Arshinoff et al., 2022); however, the spatial expression pattern of this gene in larvae has not been reported thus far. Therefore, to elucidate the expression pattern of *msi1* in sea urchin larvae, we performed whole-mount *in situ* hybridization to detect *msi1* mRNA expression. In 36-h prism larvae, *msi1* was found to be expressed exclusively in the archenteron, which is not the focus of this study (Figure 3A). In 48-h pluteus larvae, *msi1* expression was initiated in the brain and continued throughout the pluteus stage (Figures 3B, C). *msi1* was co-expressed with *foxQ2* (Figure 3D), a determinant of the brain (Yaguchi et al., 2008), but not with *rx* and *tph* at this stage (Figures 3E, F). Furthermore, *msi1* was expressed in *chat*-positive cells in the brain region (Figure 3G).

Next, we focused on the relationship between *msi1* and *rx*. As *rx* initially expresses in the same region as *foxQ2* (Figures 2B, B′), it is possible that Rx is involved in the induction of *msi1*. To investigate this, we injected Rx morpholino and examined the expression pattern of *msi1*. In Rx morphants, there was no detectable *msi1* signal in the brain (Figures 3H, I), and the percentage of *msi1*-positive larvae was 95.9% (70/73) in the control group and 3.3% (2/61) in the Rx morphant group. In conclusion, Rx regulates *msi1* transcription in the brain region of sea urchin larvae, although it remains unclear whether this control is direct or indirect.

Msi1 plays a crucial role in the expression of Chat in the larval brain and the formation of the neural plexus.

**FIGURE 4:**
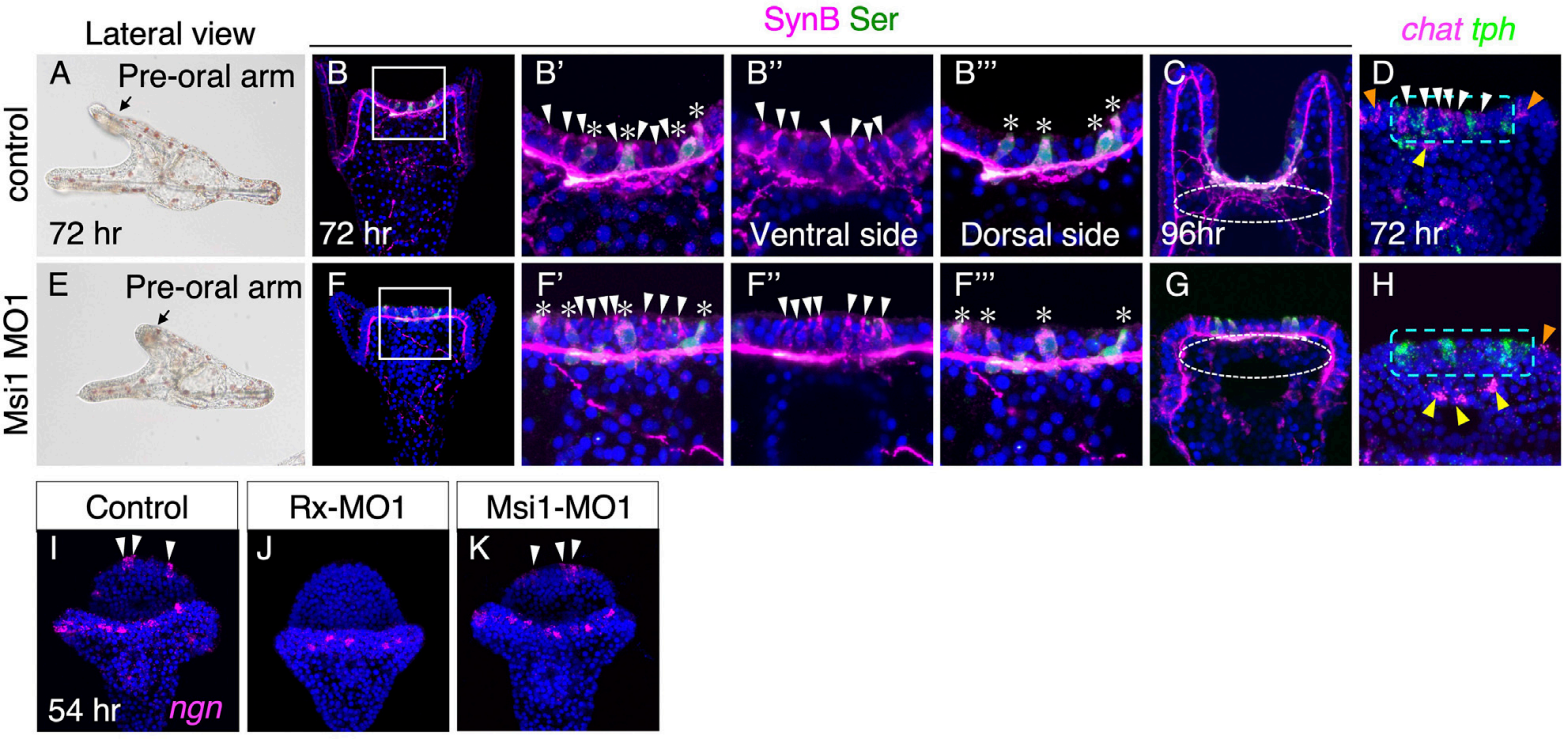
Msi1 is required for the expression of *chat*. **(A–D)** Control. **(E–H)** Msi1 morphants. **(A,E)** Bright field images of lateral view. **(B–B‴,F–F‴)** Both serotonergic (asterisks) and non-serotonergic neurons (white arrowheads) are present in Msi1 morphants as same as control. Magnified images of the square in **(B)** and **(F)**, respectively. **(C,G)** Neuro-plexus from non-serotonergic neurons are absent at 96 h in Msi1 morphant (white dotted-line circle). **(D,H)** *chat* and *tph* expression patterns. *chat* positive cells in the brain are missing in Msi1 morphants (white arrowheads). Green dot-lined rectangles show the brain region. There are *chat* positive cells of the edge of upper lip (yellow arrowheads) and ciliary band (orange arrowheads) in Msi1 morphants. **(I,J)** *ngn* positive cells (white arrowheads) are missing in the brain of Rx morphants, but not in Msi1 morphants **(K)**.

To investigate the function of Msi1, we examined the neural pattern of the brain in Msi1 morphants. Compared to the control group, Msi1 morphants exhibited shorter preoral arms (Figures 4A, E). However, similar to the control group, non-serotonergic and serotonergic neurons were present on the ventral and dorsal sides of the brain, respectively (Figures 4B–B‴, F–F‴). In contrast, the axons and dendrites from non-serotonergic neurons were rarely detected in the brain of Msi1 morphants at 96 h (dot-lined in Figures 4C, G), and the *chat* signal was absent in the brain (Figures 4D, H, Supplementary Table S1). Injecting Msi1-MO2 into embryos yielded the same phenotype as Msi1-MO1 morphants (Supplementary Figures S2A–D), providing further support for the specificity of the morpholino. These findings indicate that while non-serotonergic neurons are present in the brain of Msi1 morphants, they fail to express *chat* and extend axons and dendrites. Notably, there was no effect on the expression of *chat* in the upper lip neurons, suggesting that Msi1 is involved in the differentiation of cholinergic neurons specifically in the brain. Interestingly, when we inhibited the function of Rx, the presence of *ngn*-positive cells was significantly reduced. However, in Msi1 morpholino-treated larvae, the *ngn*-positive cells appeared to be unaffected and were observed in a normal pattern (Figures 4I–K, white arrowheads).

### The bilateral clusters on the oral side connect to the brain neurons

**FIGURE 5:**
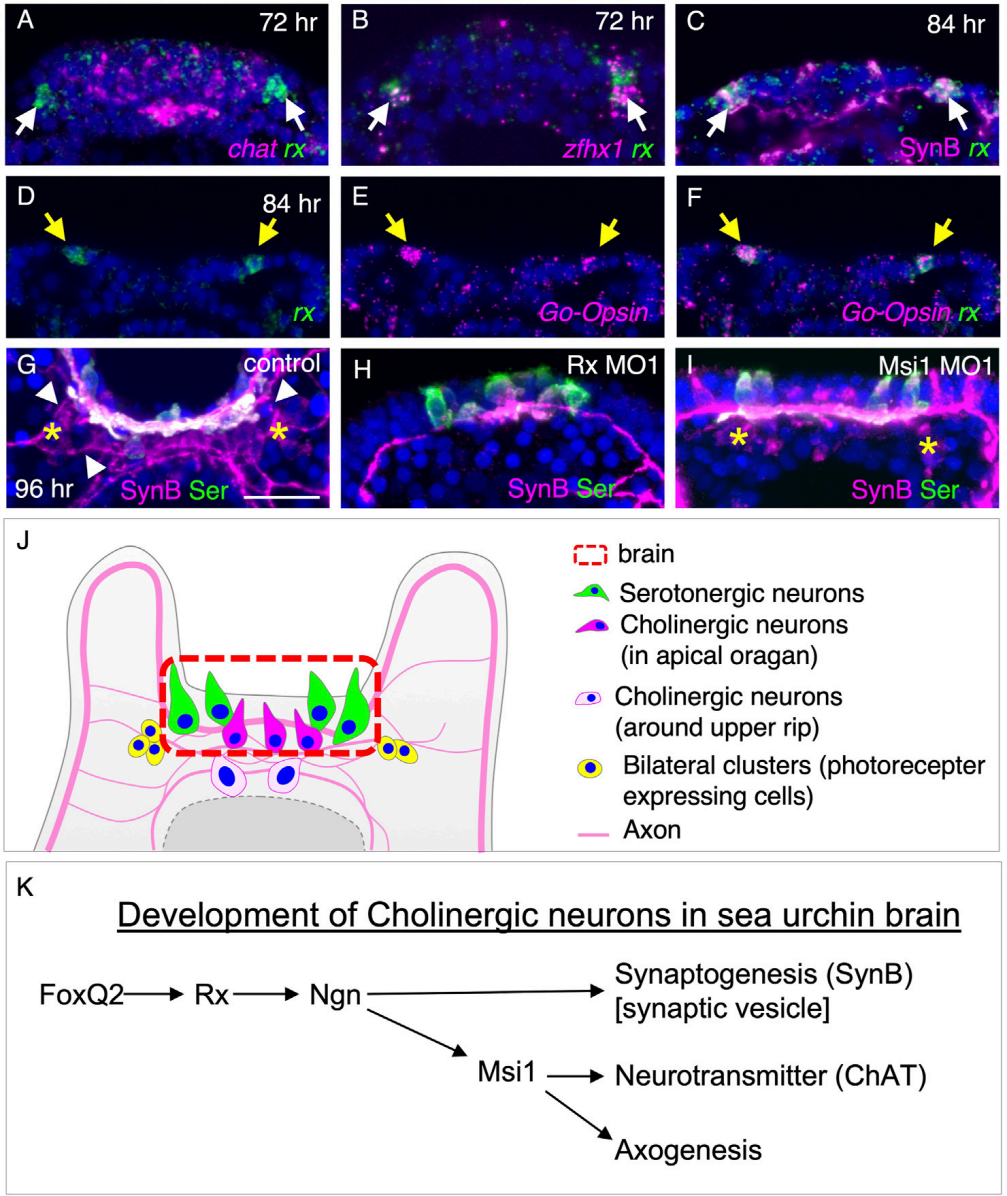
Formation of bilateral clusters on the ventral side is regulated by Rx. **(A,B,C)** *rx* expression pattern of bilateral clusters with *chat*, *zfhx1* and SynB. *rx* expressing at bilateral clusters of neurons (arrows) is not co-localized with *chat.* **(D,E,F)** *Go-Opsin* and *rx* are co-expressed in the bilateral clusters at 84 h (yellow arrows). **(G,H,I)** Bilateral clusters (yellow asterisks) is disappeared in Rx morphants but not in Msi1 morphants. Neural processes from bilateral clusters extend towards the brain neurons (arrowheads). **(J)** Schematic image of neurons in brain, the edge of the upper lip, and the bilateral clusters. **(K)** Molecular pathway for the development of cholinergic neurons in and around sea urchin brain.

To investigate the interplay between different neuronal types in and around the brain, we conducted a detailed analysis of *tph*, *chat*, *rx*, *zinc finger homeobox 1* (*zfhx1*), and *Go-opsin* expression patterns in larvae aged 48–96 h (Figure 5, Supplementary Figure S5). At 60 h, *rx* signals were observed in bilateral ventral clusters adjacent to the brain, consistent with previous findings in the sea urchin species *Strongylocentrotus purpuratus* (Valencia et al., 2021) (Supplementary Figures S5J–L). Interestingly, these *rx*-positive cells in the bilateral clusters did not co-express *chat*, a marker for cholinergic neurons (Figure 5A), but they did co-express *zfhx1*, which is known to play a role in specifying neurons in sea urchins (Yaguchi et al., 2012), as well as SynB (Figures 5B, C). Additionally, we confirmed the expression of *Go-opsin* in these *rx*-positive cells in the bilateral ventral clusters of *H. pulcherrimus* (Figures 5D–F, yellow arrows), consistent with the findings in *Strongylocentrotus purpuratus* (Valencia et al., 2021). These bilateral clusters are prominently identified in immunohistochemistry using SynB antibody (Figure 5G, yellow asterisks). To visualize the morphology of neurons, including neural processes, immunohistochemistry is much better than *in situ* hybridization since morphology of sea urchin larvae is shrunk during *in situ* hybridization treatment. Axons from these bilateral clusters are connected to brain neurons (Figure 5G, arrowheads). The bilateral clusters were disappeared in the Rx morphants, supporting the previously reported data (Valencia et al., 2021), although they were present in Msi morphants (Figures 5H, I). In addition to Valencia (2021), in which Rx morphants lost *Go-Opsin* in the bilateral clusters, the results shown here indicate that Rx is not only required for the specification of the photoreceptor clusters, but also for the characterization of them.

## Discussion

In this study, we have provided insights into the neural organization of sea urchin larvae brains (Figure 5J). While previous reports have indicated the presence of serotonergic neurons on the dorsal side and non-serotonergic (cholinergic) neurons on the ventral side of pluteus larvae brains (Yaguchi et al., 2016; Slota and McClay, 2018), it was surprising to observe their simultaneous appearance on the dorsal side, with subsequent migration of cholinergic neurons towards the ventral side during development. Although the functional significance of this patterning remains uncertain, it is expected to be important given the highly conserved neural pattern observed in each larva. The intricate complexity of neurites among these neurons suggests a higher frequency of intercommunication than previously assumed (Figure 5J). Indeed, cholinergic neurons are known to play a central role in forward swimming (Yaguchi et al., 2022), while serotonergic neurons control anti-gravity responses (Yaguchi and Katow, 2003). Notably, exposure to intense light inhibits the function of cholinergic neurons, resulting in the loss of anti-gravity swimming behavior. These findings strongly suggest that the sea urchin brain processes environmental signals and mediates them to larval behaviors, which are coordinated by the cilia distributed throughout the body (Yaguchi et al., 2022). However, due to current technological limitations, the precise connectivity between individual neurons and the mechanisms of signal transmission in sea urchin nervous systems remain unclear. Further investigations, such as connectome studies or neurophysiological analyses, hold promise for unveiling the detailed neurological mechanisms underlying brain function in sea urchin larvae.

This study provides the first evidence of neuronal shifting and changing locations in the brain region of echinoderms. In the cerebral cortex of vertebrate brains, the cell bodies of newborn neurons undergo migration between the ventricular zone and marginal zone during development. Failure in precise migration and positioning can lead to impaired brain function (Ayala et al., 2007). While this work did not trace the individual cell shifts, we cannot conclusively determine whether these shifting neurons migrate among epithelial layers or change their relative positions within the brain. By tracing the lineage of a single blastomere from the 8-cell stage, we observed that some labeled neurons were entirely absent in non-labeled regions of the brain. This finding suggests that these neurons do not actively migrate in sea urchin larvae. Therefore, it is strongly indicated that individual neurons rarely undergo independent migration but instead change their relative locations through cell division within the ectoderm. It has been reported that ciliary band neurons undergo small-distance migration in sea urchin larvae, thereby suggesting a developmental sequence similar to that observed in neural crest cells of vertebrates (Slota et al., 2020). While the Ambulacrarian clade lacks the neural crest cell lineage, the accumulation of molecular data on migratory neurons will provide valuable insights into the emergence of neural crest cells within the deuterostome group during evolution.

Detailed observations in this study have revealed the presence of upper lip cholinergic neurons in close proximity to the brain. These neurons extend their neurites towards brain neurons, indicating potential communication, particularly during eating behaviors. It is evident that the developmental regulation of these upper lip neurons differs from that of brain neurons. Notably, the upper lip neurons remain unaffected in Rx- or Msi1-morphants (as observed in this study), whereas brain neurons show some effects in response to these manipulations. Furthermore, the formation of upper lip neurons is regulated downstream of the dorsal-ventral axis, which is controlled by the Nodal/BMP pathway (Duboc et al., 2004). In embryos injected with ∆cadherin, where the brain region occupies the entire body and the Nodal/BMP-specified ectoderm is absent, the upper lip neurons are absent as well (Logan et al., 1999). However, cholinergic neurons within the expanded brain region are still present, albeit with imprecise patterning. This discrepancy suggests that the dorsal-ventral patterning of the brain region is heavily reliant on the Nodal/BMP pathway (Yaguchi et al., 2016).

Rx is a prominent transcription factor known for its role in regulating eye specification in vertebrates. However, in eyeless organisms such as sea urchins, the function of Rx has remained enigmatic despite its reported expression in various species. This study sheds light on the requirement of Rx in the specification of photoreceptor cells. Intriguingly, Rx also plays a role in ocellus development in ascidians, indicating a conserved function related to photoreceptors among deuterostomes (Aniello et al., 2006). This suggests that the function of Rx in photoreceptor development was established in the common ancestor of deuterostomes. Furthermore, Rx is essential for eye formation in protostomes, including annelids, supporting the notion that the function of Rx is conserved throughout bilaterians, as previously proposed (Tessmar-Raible et al., 2007). However, in ecdysozoans like fruit flies, Rx is not necessary for the establishment of the visual system, posing challenges in the discussion of the evolution of Rx function in photoreceptor system development (Eggert et al., 1998; Viets et al., 2016). Additionally, the independent function of Rx in cnidarians, which is unrelated to photoreceptor cell formation, further highlights the conservation of photoreceptor-related Rx function within the deuterostome clade (Kon and Furukawa, 2020).

Our findings demonstrate the essential role of Rx in the differentiation of neurons, particularly cholinergic neurons, and their axon development in the sea urchin larval brain. Interestingly, the specification of the brain field, as indicated by the pattern of serotonergic neurons, was unaffected in Rx morphants, suggesting that the dorsal-ventral and left-right axes of brain region were preserved. In contrast, Hbn morphants exhibited a completely opposite phenotype, where the size of the brain remained unchanged but serotonergic neurons were absent. Notably, Rx and Hbn are adjacent genes in the sea urchin genome (Sodergren et al., 2006; Kinjo et al., 2018; Arshinoff et al., 2022), as observed in other organisms (Mazza et al., 2010), and their initial expression patterns in the entire prospective brain region completely overlapped (Figure 2C). This strongly suggests shared transcriptional regulation between these two genes, with both likely acting downstream of brain field initiators such as FoxQ2 (Figure 5K). The co-expression and complementary functions of these genes in neural development are particularly intriguing. Furthermore, our study provides valuable insights into axon development in sea urchin larvae, as the involvement of Rx expands our understanding of how neural networks are established in these organisms, an area that has been largely unexplored.

Our data shows that the regulation of axon development by Rx is mediated by Msi1 in sea urchin larvae. The expression of *msi1* in the brain region occurs significantly later than *rx* expression, suggesting that early Rx may indirectly control Msi1 transcription probably through Ngn (Slota and McClay, 2018) (Figure 5K), or that the Rx protein persists in the brain for an extended period, regulating the transcription of downstream genes, including Msi1. Msi1 is a critical gene involved in neurogenesis in various organisms. For instance, it plays a role in fate determination of dividing neural progenitors in fruit flies and mediates axon development through post-transcriptional regulation of Robo3/Rig-1 in mice (Nakamura et al., 1994; Sakakibara et al., 2001; Okano et al., 2002; Kuwakoichiro et al., 2010). In our study, we discovered that Msi1 is required for the expression of *chat* in non-serotonergic neurons of sea urchin brains (Figure 5K). Based on their morphology, the non-serotonergic neurons in Msi1 morphants undergo terminal differentiation but fail to express the *chat* gene. Additionally, their neural processes extend only along the left-right body axis. Currently, we do not have information about the specific neurotransmitters or neuropeptides produced by these neurons other than acetylcholine in *H. pulcherrimus*. Furthermore, the absence of a neuroplexus on the ventral side in Msi1 morphants suggests that Msi1 is crucial for axon development in sea urchins (Figure 5K). Although the spatial expression pattern of Robo homologs remains unclear, their temporal expression coincides with the stage of pluteus, where brain axon development would occur. It will be intriguing to investigate whether the functional pathway of Msi1 in sea urchins is similar to that in mice. Future expression analyses, such as *in situ* hybridization of Robo or single-cell RNA-seq, along with functional assays in *H. pulcherrimus*, will unveil the detailed molecular pathway of Msi1 in the brain region of sea urchin larvae.

In summary, our findings provide evidence of the conserved expression pattern and functional role of Rx in echinoderm larvae among deuterostomes, suggesting its importance in brain and photoreceptor development across clade. Rx exhibits an initial expression in the prospective brain region, followed by its expression in photoreceptor cells. Its primary function in the brain appears to be involved in specific aspects of neural differentiation, such as axon development, rather than regional brain specification, as depicted in Figure 5K. As downstream of the regional specifier, FoxQ2, Rx functions in promoting neural differentiation probably through Ngn (Slota and McClay, 2018) (Figure 4). Rx modulates the expression of synB independently of Msi1, while also playing a crucial role in the precise regulation of chat expression and axogenesis through Msi1 function (Figure 5K). Subsequently, Rx contributes to the development of photoreceptor cells. These evolutionary conservations involving Rx in the brain and photoreceptors raise intriguing questions regarding the evolution of light-sensing behaviors. Despite being eyeless invertebrate deuterostomes, echinoderms like sea urchins, these findings underscore the mystery surrounding how the ancestors of vertebrates acquired visual eyes and the accompanying information-processing brain.

## Data Availability

The original contributions presented in the study are included in the article/Supplementary Material, further inquiries can be directed to the corresponding author.

## Abbreviations

Rx, retinal homeobox; SynB, Synaptotagmin B; TPH, tryptophan 5-hydroxylase; DAPI, 4’,6-diamidino-2-phenulindole; ChAT, choline acetyltransferase; PBS, phosphate buffered saline; MO, morpholino oligonucleotide; RT, room temperature; HRP, horseradish peroxidase.
