# Supplementary material for: Rx and its downstream factor, Musashi1, is required for establishment of the apical organ in sea urchin larvae
Source: Front Cell Dev Biol. 2023 Aug 15;11:1240767. doi: 10.3389/fcell.2023.1240767 (PMC10465340; doi:10.3389/fcell.2023.1240767)
Supplement: Supplementary file 1 [file DataSheet1.PDF]

Supplemental Material

Rx and its downstream factor, Musashi1, is required for establishment of the apical organ in sea urchin larvae.

Junko Yaguchi, Shunsuke Yaguchi*

*** Correspondence:** yag@shimoda.tsukuba.ac.jp

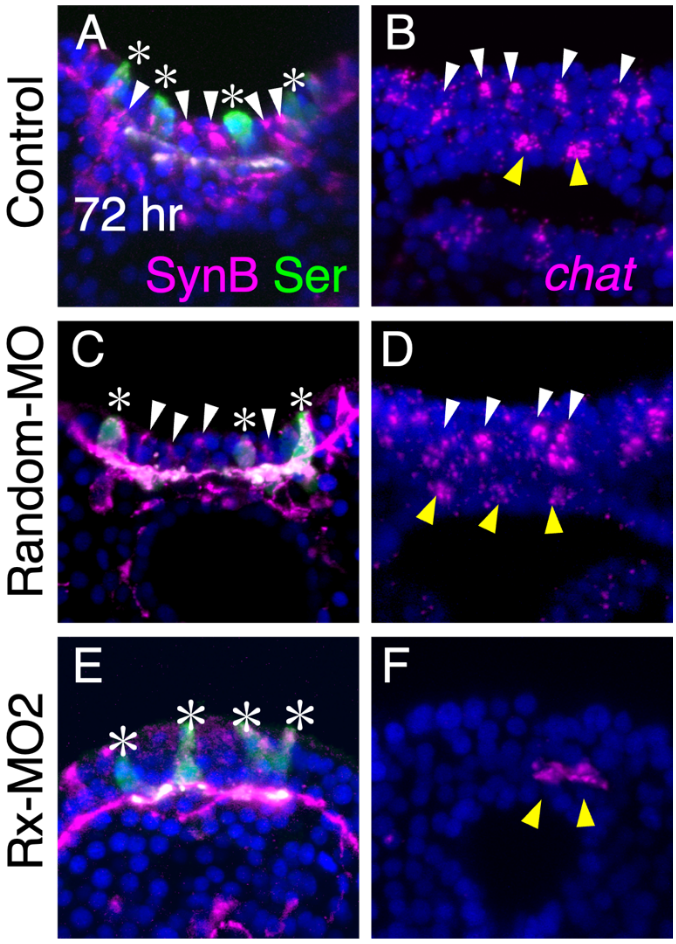

Figure S1. Specificity of Rx morpholino antisense oligo. Control (A, B; buffer-injected) and random morpholino injected (C, D) have normal nervous system in their brains. On the other hand, Rx-MO2 injected embryos lost non-serotonergic neurons (white arrowheads; cholinergic) in the brain (E,F). Asterisk, serotonergic neurons, yellow arrowheads *chat*- positive neurons at upper lip.

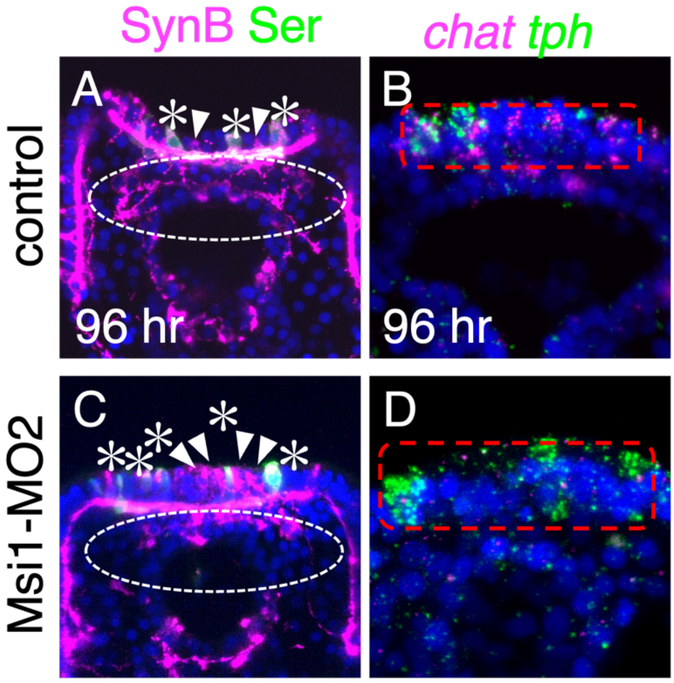

Figure S2. Specificity of Msi1 morpholino antisense oligo. (A, C) Serotonergic neurons (asterisks) and non-serotnergic neurons (arrowheads; cholinergic) are invariant in control and Msi1-MO2 injected larvae. However, the expression of *chat* (B, D) and axogenesis (A, C; white dot-lined circle) are missing in Msi1-MO2 larvae as in Msi1-MO1 morphants. Red lined rectangle indicates brain region in (B, D).

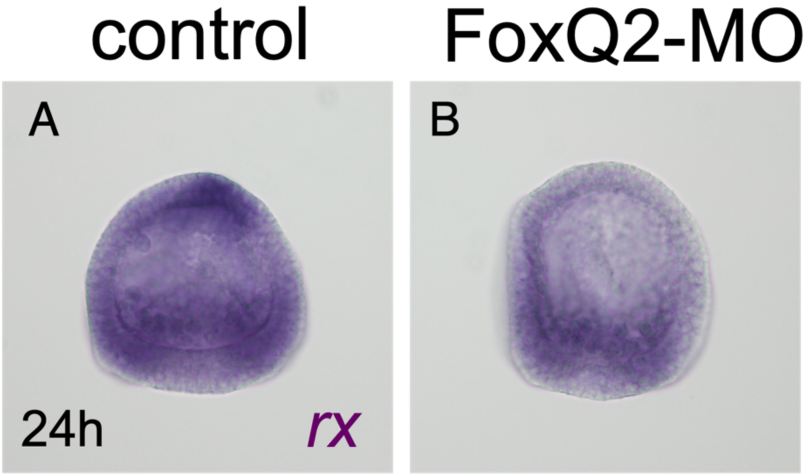

Figure S3. Rx is a downstream factor of FoxQ2. (A, B) Rx is expressed at the brain region in control (A), but is absent in FoxQ2 morphants (B).

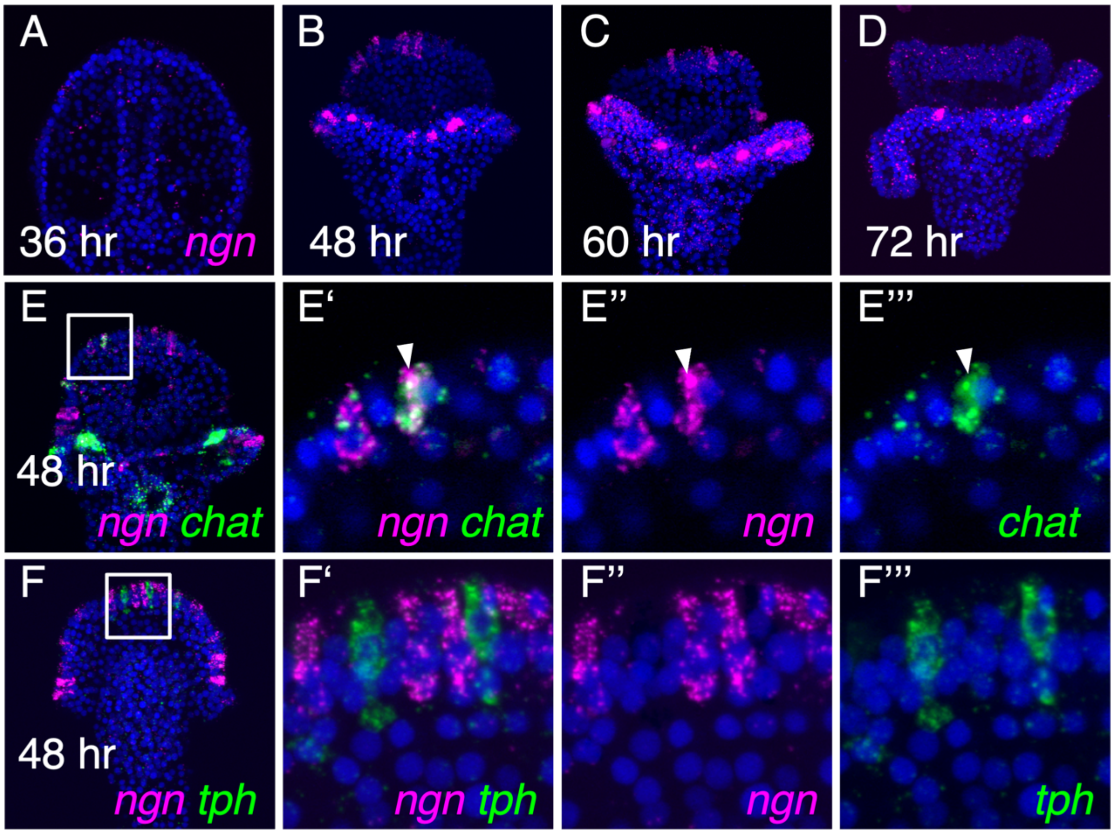

Figure S4. *ngn* expression pattern in *H. pulcherrimus*. (A, B, C, D) *ngn* expression at brain region is detected from 48 hr to 60 hr. (E – E''') *chat* is co-expressed with *ngn* (white arrowhead). (E'-E''') Magnified image of a square region in (E). (F – F''') *tph* is not co-localized with *ngn*. (F'-F''') Magnified image of a square region in (F).

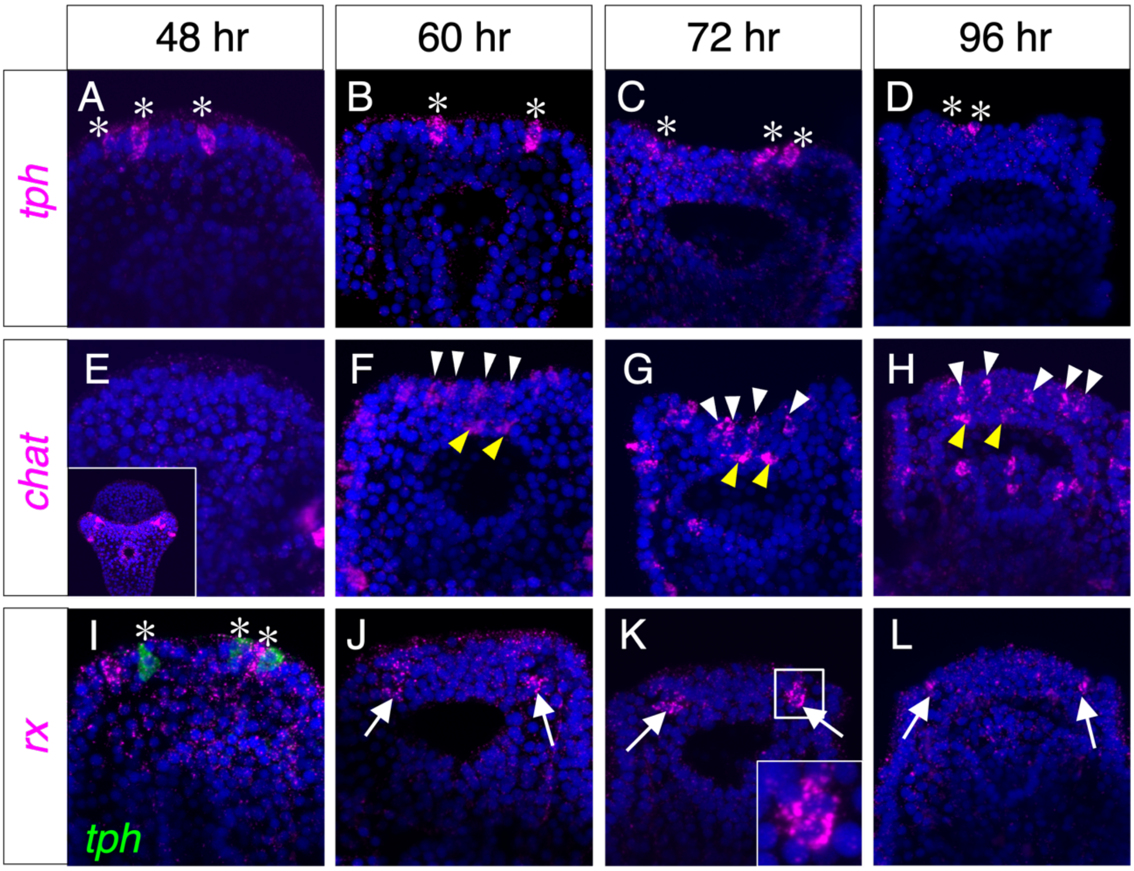

Figure S5. Expression patterns of *tph*, *chat*, *rx*, and *opsin3.2* in and around brain. *tph* (A-D), *chat* (E-H), and *rx* (I-L) expression patterns from 48 hr to 96 hr. Inset in E shows whole-body. Inset in K shows the magnified image of the square. Asterisks, serotonergic neurons; white arrowheads, cholinergic neurons in brain; yellow arrowheads, cholinergic neurons in upper lip; arrows, bilateral clusters on the ventral side.

Table S1.

	Serotonin expressed cells (N = 4)	*chat* expressed cells (N = 5)
control	7.0 ± 0.37 (n=22)	5.5 ± 0.28 (n=18)
Rx-MO1	6.2 ± 0.49 (n=19)	0.2 ± 0.13 (n=16)
Msi1-MO1	6.4 ± 0.45 (n=17)	0.8 ± 0.27 (n=13)

The quantification of serotonergic and cholinergic neurons within specific brain regions was performed in control specimens, Rx morphants, and Msi1 morphants. The numbering of cell populations was conducted by analyzing sequential optical sections of each sample (n) acquired via confocal microscopy
